# A dataset of subjectivity classification in Indonesian ride-hailing app reviews

**DOI:** 10.1016/j.dib.2025.112348

**Published:** 2025-12-01

**Authors:** Violeta Arifin, Yuriashi Adelia Putri, Richard Wiputra

**Affiliations:** Information Systems Department, School of Information Systems, Bina Nusantara University, Indonesia

**Keywords:** Subjectivity detection, Manual annotation, Binary classification, Ride-hailing, Indonesian-language reviews, Large language model (LLM), Human feedback interpretation

## Abstract

As more people share their experiences online, understanding whether their reviews are subjective or objective has become key to evaluating how services are perceived, especially in the Indonesian ride-hailing industry. This article presents a subjectivity dataset of 1338 Indonesian-language ride-hailing app reviews collected from the Google Play Store. To enhance the quality and consistency of the data for analysis, all reviews were preprocessed to eliminate elements such as URLs, emojis, and extraneous characters. Two independent annotators manually annotate the dataset, followed by a consensus-based adjudication process to produce a high-quality classification. The annotation supports robust evaluation of subjectivity detection models and contributes toward developing more nuanced natural language understanding systems in low-resource languages. The data can be reused for multiple research purposes, including benchmarking supervised classifiers, evaluating multilingual and large language models, analyzing cross-domain generalization, and extending subjectivity detection research to other Southeast Asian languages. The dataset also serves as a high-quality reference resource due to its structured annotation design and consensus-based labeling procedure, which enable reproducible analysis across different modeling approaches. By offering a transparent and fully documented dataset, this work provides a valuable foundation for developing intelligent systems capable of interpreting user feedback in real-world digital service environments, particularly in the Indonesian ride-hailing sector.

Specifications Table

**Table:** 

Subject	Computer Sciences
Specific subject area	This dataset is developed for binary subjectivity classification tasks in Indonesian-language ride-hailing app reviews. The dataset is designed to support advanced Natural Language Processing (NLP) applications and identify subjective and objective language in user-generated content. Two human annotators annotate each review to ensure consistency and reliability. This binary classification (Subjective or Objective) is crucial for understanding customer perception, filtering factual complaints, and enhancing user feedback systems in digital transportation platforms. Insights derived from this dataset can be used for service quality monitoring, automated moderation, and improved customer support in Indonesia's growing ride-hailing market.
Type of data	Annotated, Raw, Filtered, Analyzed
Data collection	The dataset was constructed by scraping user reviews from leading Indonesian ride-hailing app platforms (Gojek, Grab, Maxim) using web scraping tools.
Data source location	The reviews were collected and processed at Bina Nusantara University, Indonesia. Primary Data Source: Google Play Store – ride-hailing apps including Gojek, Grab, and Maxim.
Data accessibility	Repository name: Mendeley Data Data identification number: 10.17632/3gsbkm8p8r.2 Direct URL to data: https://data.mendeley.com/datasets/3gsbkm8p8r/2
Related research article	none

## 1 Value of the Data

• This dataset comprises 1338 annotated ride-hailing app reviews sourced from Gojek, Grab, and Maxim, three major Indonesian ride-hailing platforms. It provides a unique resource for analyzing subjectivity detection in user reviews. The dataset distinguishes between subjective and objective content in user-generated feedback, giving valuable insights into user sentiment. This dataset is essential for various NLP tasks, such as sentiment analysis, opinion mining, and feedback classification, which can help better understand and process user opinions.
• This dataset consists entirely of reviews in Bahasa Indonesia, a language that remains relatively underrepresented in NLP, which contributes to the development of resources for low-resource languages. Its structured annotation guideline and clearly defined task formulation make it suitable for training and benchmarking machine learning and deep learning models tailored to Indonesian-language applications.
• This dataset captures natural context and organically user-generated feedback from real-world ride-hailing app reviews on the Google Play Store, which provides authentic linguistic patterns that enhance the ecological validity of models trained on it. By reflecting the dynamic nature of Indonesia’s rapidly evolving ride-hailing sector, the dataset also offers a foundation for future expansion to additional platforms, ensuring continued relevance for research on user behavior and digital mobility services.
• This dataset can be reused in multiple future research directions, including benchmarking subjectivity detection models, evaluating cross-domain generalization, exploring transfer learning to related classification tasks, and assessing the performance of large language models under different prompting strategies. Built with structured annotations and a clear task definition, this dataset provides a reliable benchmark for subjectivity detection in Bahasa Indonesia and supports consistent comparison across machine learning and deep learning approaches.
• Compared with existing Indonesian benchmark datasets such as IndoNLU, which include broad NLU tasks across diverse domains, this dataset focuses on a specialized and underexplored task: subjectivity detection in ride-hailing app reviews. Rather than overlapping with IndoNLU, it complements and extends the current landscape of Indonesia NLP resources by introducing a domain-specific, task-focused dataset that captures a real-world application scenario.
• This dataset has limitations, particularly the absence of demographic metadata (e.g., age, gender, region) and detailed temporal metadata. These limitations restrict its suitability for sociolinguistic analyses or studies examining variations across user groups or time. Future work may incorporate such metadata to support richer interdisciplinary research.

## 2 Background

The development of this dataset was driven by the need to support research in subjectivity detection within ride-hailing app reviews. As the ride-hailing sector is one of the four leading sectors in Indonesia that significantly impacts the growth of the internet economy [1], understanding the nuances of user feedback is essential for enhancing service quality and user satisfaction. Despite the sector’s prominence, research in subjectivity detection for Bahasa Indonesia remains limited, and no publicly available dataset has previously focused on this task within transportation services. Existing Indonesian NLP resources such as IndoNLU do not cover subjectivity detection tasks, which creates a clear gap for this specific classification task. This dataset addresses this gap by providing a curated and manually annotated collection of 1338 user reviews across Indonesian ride-hailing platforms, including Gojek, Grab, and Maxim. It was annotated by two independent human annotators, classifying them as either Subjective (1) or Objective (0), following predefined labeling guidelines. It is also the first publicly available subjectivity dataset in Bahasa Indonesia that reports inter-annotator agreement which strengthens its reliability as a research resource. This dataset plays a crucial role in subjectivity detection tasks and developing models tailored to the linguistic and cultural features of Bahasa Indonesia. It helps create more advanced NLP applications with a strong capability to interpret and process the unique aspects of the language effectively. Future research can use this dataset as a foundation by expanding its focus to the broader urban mobility sector for a more comprehensive understanding of user perspectives in Indonesia’s rapidly evolving digital transportation landscape.

## 3 Data Description

The dataset was collected from user reviews on Indonesian ride-hailing applications such as Gojek, Grab, and Maxim. This dataset focuses on the ride feature to standardize the comparison across all three applications, representing a core and commonly available service. This dataset reflects real-world feedback from app users, offering valuable insight into customer perceptions and subjective patterns within the Indonesian digital transportation sector.

Gojek, founded in 2010 by Nadiem Makarim, began as a call center with 20 motorcycle taxis in Jakarta [2]. Over the years, it evolved into a leading super app in Southeast Asia, launching its platform in 2015 with services like transportation (GoRide and GoCar), deliveries (GoSend), shopping (GoShop), food (GoFood), and digital payments (GoPay) [3]. By 2016, Gojek became Indonesia's first unicorn, and by 2018, it expanded to Vietnam and Thailand, with daily transactions reaching 100 million [4]. Gojek operates in over 200 cities across Indonesia, Vietnam, Thailand, and Singapore, with a fleet of more than 2 million drivers and delivery partners [4]. GoPay, Gojek's digital payment service, is accepted by over 900,000 merchants and remains dominant in Indonesia’s digital economy [5]. In 2023, GoPay was recognized as the "Best App" in the Google Play Awards, with a study showing 71 % of Indonesian digital wallet users have tried it, and 58 % use it regularly, reflecting its strong market presence and loyalty [6,7].

Initially launched as MyTeksi in Malaysia in 2012, Grab has transformed into Southeast Asia's leading super app [8]. Headquartered in Singapore, Grab offers a comprehensive suite of services, including ride-hailing, food delivery, digital payments, and financial services across eight countries: Singapore, Malaysia, Cambodia, Indonesia, Myanmar, the Philippines, Thailand, and Vietnam [8]. Operating in over 500 cities across eight countries, Grab has become one of Southeast Asia’s most valuable firms, with a market of $12 billion [9]. In Indonesia, Grab has tailored its services to local preferences, resulting in over 20 million active users for its motorcycle taxi services [8]. The company's financial arm, OVO, stands as the most frequently used e-wallet in Indonesia, supporting transactions with over 1.5 million QRIS merchants across 600 cities in Indonesia and enhancing digital payment accessibility nationwide [10,11].

Maxim is a Russian technology company that operates taxi aggregation and food tech businesses and offers additional services such as delivery and cargo [12]. Founded in 2003, Maxim entered the Indonesian market in 2018 and has since expanded its services to over 100 cities nationwide [13]. Maxim provides diverse services, including ride-hailing, delivery and purchase of food or goods, cleaning, laundry, and so on [12]. It is widely recognized for its cost-efficiency, offering more competitive pricing than other ride-hailing services in the region [14]. In 2024, Maxim launched its digital wallet, KasPro, licensed by Bank Indonesia. It enables secure financial transactions for rides and services payment, balance top-ups via mobile banking or in-person methods, and driver tipping, aligning with Indonesia's growing adoption of digital payments [15]. As of 2025, Maxim continues to serve millions of users in Indonesia, competing with other ride-hailing services in the region.

This study presents a detailed dataset analysis focused on subjectivity detection within Indonesian ride-hailing app reviews, comprising 1338 samples from three prominent ride-hailing platforms, Gojek, Grab, and Maxim. Unlike many existing datasets that primarily focus on general sentiment analysis, our dataset addresses distinguishing subjective and objective content in reviews written in Bahasa Indonesia. For example, the dataset discussed in [16] is quite a large scale of reviews with 10.806 samples in Bahasa Indonesia classified into three labels: positive, negative, and neutral, but it only focuses on general sentiment analysis in Twitter. A dataset [17] also explores sentiment analysis in greater depth by categorizing emotional polarity into specific emotions such as love, joy, anger, sadness, and fear; however, it still relies on data sourced from Twitter. Similarly to our dataset, the dataset used in [18] also comprises user reviews from a ride-hailing app, but it is limited to a single platform, Gojek. It also focuses on sentiment analysis, which is classified into four categories: very positive, moderately positive, moderately negative, and very negative. These comparisons highlight the broader and distinct scope of our dataset, which uniquely focuses on detecting subjectivity in Indonesian language reviews across three ride-hailing apps as an area that has not been addressed explicitly in previous studies.

In developing this dataset, we implemented a two-step annotation process to ensure high-quality and reliable labels for subjectivity detection tasks. In the first stage, the data were manually labeled by two human annotators independently as subjective or objective based on predefined labeling guidelines, resulting in disagreements highlighting the inherent complexity of subjectivity detection in user reviews (shown in Table 1). Therefore, the annotators carried out a discussion as the second step to resolve disputes and finalize the labels to enhance the dataset's quality and suitability for subjectivity detection tasks in ride-hailing app reviews (shown in Table 2).

**Table 1:** Overview of sample data and column structure in the dataset per annotator CSV.

Score	App	Review	Translated_review	1st_annotator_label	2nd_annotator_label
1	Grab	kurang suka di cara pemilihan peta nya yang tidak bisa di atur dengan akuratmasa harus pasang pin di tempat yang harus ada di maps	Don’t like the map selection method that can’t be adjusted accurately, must put pin on a place that must be on maps	1	0
1	Maxim	kecewa berat sih percuma punya fitur ewallet klo smua drivernya di kota makssr gk ada yg make tolong fitur ewalletnya di fungsiin ya kak biar gak kalah saing sm kompetitor sblh	Very disappointed, useless to have e-wallet feature if all drivers in Makassar city don’t use it, please activate e-wallet feature so we don’t lose to competitors	1	1
5	Gojek	alhamdulillah selalu memuaskan pake aplikasi gojek menolong banget di saat sy ada di tempat yg asing tapi alhamdulillah bisa sampe ke tempat tujuan dianter abang gojek	Alhamdulillah always satisfied with Gojek app, very helpful when I’m in unfamiliar places, can reach destination thanks to abang Gojek	1	1
1	Gojek	kendala tidak menemukan driver tp sistemnya gak ada solusi bertanya ke pusat bantuan juga sama saja tidak bisa dikasih masukan	Problem can’t find driver but system has no solution, asking help center same, can’t give feedback	0	1
3	Gojek	jadi takut kalo pake voucher hemat disinikalo pakenanti driver nya judesngasih rating customer nya jelekjadi susah dapet driver ga dikasih tips pasti ngomel	Scared to use voucher hemat here because drivers are rude and give bad ratings to customers, hard to get driver, if no tip they complain	1	1

**Table 2:** Overview of sample data and column structure in the dataset final CSV.

Score	App	Review	Translated_review	Label
1	Grab	kurang suka di cara pemilihan peta nya yang tidak bisa di atur dengan akuratmasa harus pasang pin di tempat yang harus ada di maps	Don’t like the map selection method that can’t be adjusted accurately, must put pin on a place that must be on maps	0
1	Maxim	kecewa berat sih percuma punya fitur ewallet klo smua drivernya di kota makssr gk ada yg make tolong fitur ewalletnya di fungsiin ya kak biar gak kalah saing sm kompetitor sblh	Very disappointed, useless to have e-wallet feature if all drivers in Makassar city don’t use it, please activate e-wallet feature so we don’t lose to competitors	1
5	Gojek	alhamdulillah selalu memuaskan pake aplikasi gojek menolong banget di saat sy ada di tempat yg asing tapi alhamdulillah bisa sampe ke tempat tujuan dianter abang gojek	Alhamdulillah always satisfied with Gojek app, very helpful when I’m in unfamiliar places, can reach destination thanks to abang Gojek	1
1	Gojek	kendala tidak menemukan driver tp sistemnya gak ada solusi bertanya ke pusat bantuan juga sama saja tidak bisa dikasih masukan	Problem can’t find driver but system has no solution, asking help center same, can’t give feedback	1
3	Gojek	jadi takut kalo pake voucher hemat disinikalo pakenanti driver nya judesngasih rating customer nya jelekjadi susah dapet driver ga dikasih tips pasti ngomel	Scared to use voucher hemat here because drivers are rude and give bad ratings to customers, hard to get driver, if no tip they complain	1

**Fig. 1:**
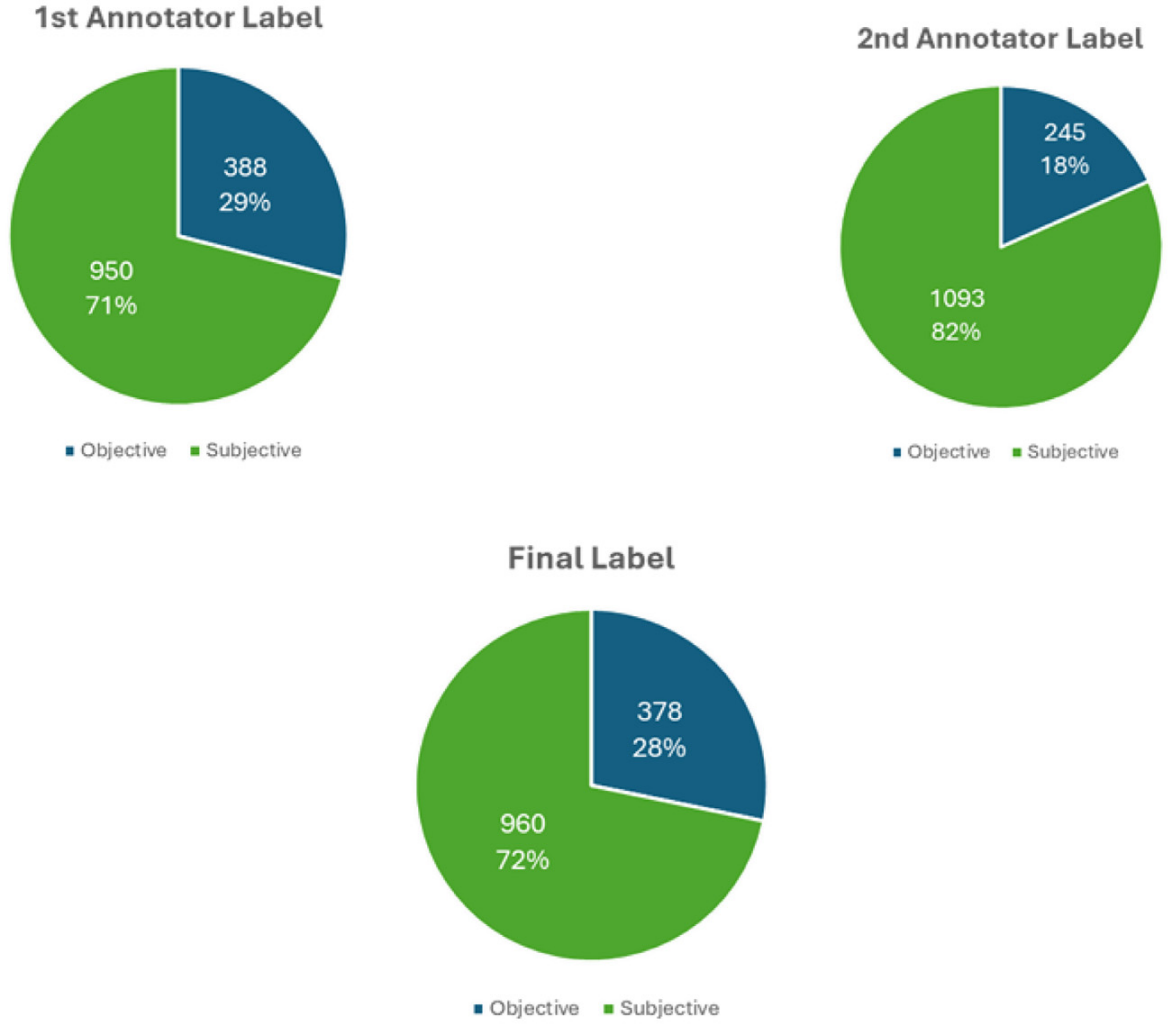
Annotator agreement and label distribution in subjectivity detection.

This dataset underwent manual labeling by two independent annotators to classify the reviews as subjective or objective based on predefined labeling criteria. The first annotator labeled 950 out of 1338 reviews (71 %) subjective and 388 (29 %) objective. The second annotator labeled the same dataset, identifying 1093 reviews (82 %) as subjective and 245 (18 %) as objective.As shown in Fig.1, subjective content predominates in the user reviews. After resolving annotation discrepancies, the finalized labels comprise 960 subjective reviews (72 %) and 378 objective reviews (28 %), establishing a reliable benchmark for subjectivity detection research in the transportation domain.

The dataset is stored in two CSV files: ‘Subride - Subjectivity Detection in Ride-Hailing App Reviews Per Annotator.csv’ and ‘Subride - Subjectivity Detection in Ride-Hailing App Reviews Final.csv’. These files contain detailed annotations and reviews for subjectivity detection in ride-hailing app reviews. The columns in the files are structured as follows:

1. Score: The rating the user gives for the review on the respective ride-hailing app (from 1 to 5).
2. App: Specifies the ride-hailing app from which the review was sourced, such as Gojek, Grab, or Maxim
3. Review: The actual content of the user’s review, which has undergone preprocessing to remove URLs, emojis, and unnecessary characters, ensuring that the text is clean and suitable for research purposes.
4. Translated_review: The English translation of the original user review. All translated reviews align with the preprocessed version of the original Indonesian review.
5. 1st_annotator_label: The label assigned by the first annotator, indicating whether the review is subjective or objective.
6. 2nd_annotator_label: The label assigned by the second annotator also classifies the review as subjective or objective.
7. Label: The final consensus label, either subjective or objective, was reached after a discussion between the two annotators to resolve disagreements and achieve an agreement.

## 4 Experimental Design, Materials, and Methods

### 4.1 Data collection

**Fig. 2:**
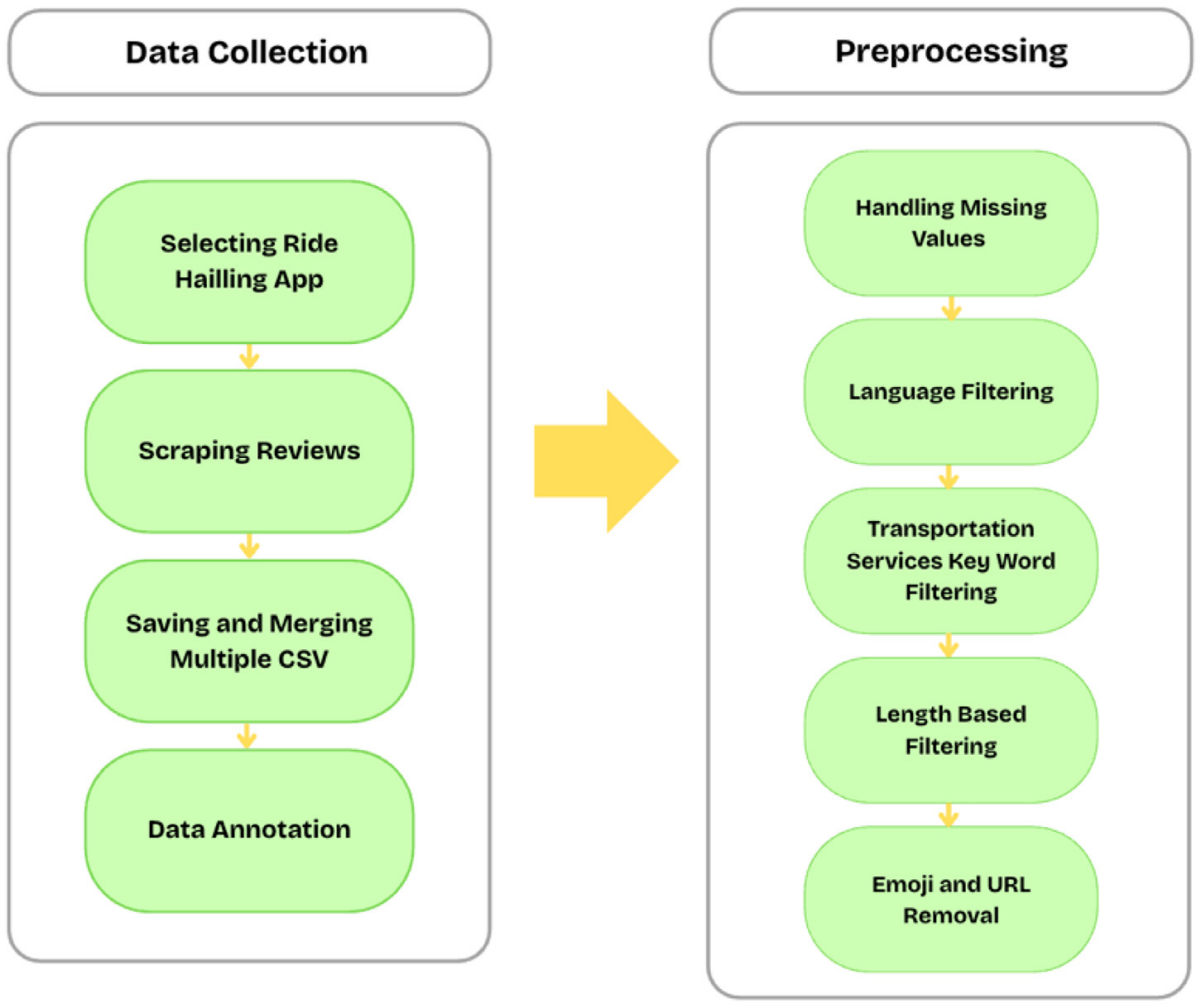
Flowchart for data collection and annotation.

The data collection used in this study contains several steps, including the data collection and preprocessing steps, which are shown in a detailed flowchart in Fig. 1. The initial steps include selecting ride-hailing apps from which the data will be collected. Following this, we proceed to scrape the reviews. Once the reviews were collected, we performed the preprocessing tasks. This phase included the removal of Nan values, emojis, URLs, keyword filtering, and length-based filtering. The data will only be filtered to Bahasa Indonesia reviews, and keyword filtering will only be used for transportation service-related reviews. The final phase of the data collection process involved manual annotation. This manual annotation phase was crucial for accurately categorising each review as subjective or objective. These comprehensive phases from initial selection to annotation were illustrated in Fig. 2.

**Fig. 3:**
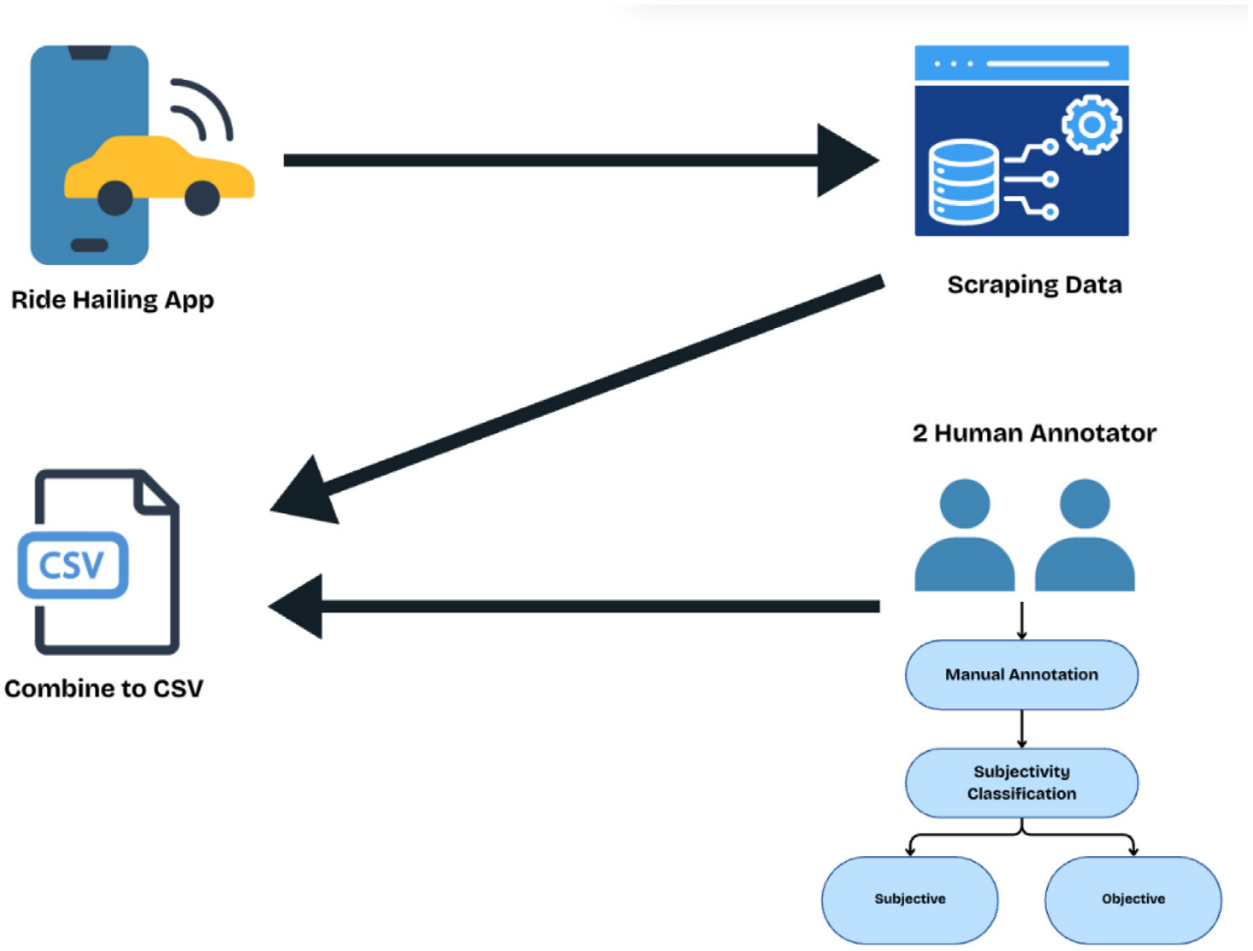
Data scraping and annotation process.

The figure provided summarizes our systematic approach to data collection and annotation. A detailed summary of the structured process is provided below:

• Selection of ride-hailing app: In the initial stage, major Indonesian ride-hailing platforms were chosen, including Gojek, Grab, and Maxim. These apps are widely used in Indonesia, providing transportation-related services. This stage was crucial because it ensured the data used in this study captured the broad spectrum of customer opinions related to ride-hailing apps.
• Web Scraping Process: In the second stage, data were collected exclusively from the Google Play Store, covering the period from November 2024 to March 2025. The scraping process was conducted using the Google Play Scraper tool, operated within the Visual Studio Code (VS Code) environment. Fig. 3 illustrates the methodology used for data collection, encompassing the selection of ride-hailing apps and the processes of annotation and scraping. For each selected application, the collected data included the following fields:
  ○ Name: the username of the reviewer.
  ○ Score: the rating assigned by the user to the app.
  ○ App: the name of the application from which the review was obtained.
  ○ Review: the textual content of the user's feedback.

However, the 'Name' field was removed from the final dataset to maintain user privacy and comply with data protection standards.

Filtering and Preprocessing Data: In the third stage, the collected data underwent several filtering and preprocessing steps to improve its quality. Following the scraping process, a significant amount of noisy and irrelevant data was detected in the dataset. We removed entries with missing values (NaN) to address this and ensure that all reviews contained meaningful content. Since the dataset focuses on Bahasa Indonesia reviews, we filtered the data to retain only reviews written in Bahasa Indonesia, maintaining language consistency throughout the dataset. Next, we applied keyword-based filtering to include only reviews related to transport services and exclude reviews concerning other services; the keywords used for filtering are listed in Table 3. We then implemented a length-based filtering step, selecting only reviews with at least 15 words to prioritise more informative feedback. Finally, we removed emojis and URLs from the reviews to clean the dataset further, resulting in a cleaner and more standardised collection of text for subsequent analysis.

• Data Annotation: In the final step, the manual annotation for subjective and objective was added in the 'label' column. Two annotators manually annotated each review based on the predetermined labeling guideline.

**Table 3:** Keywords used for transportation-related review filtering.

Category	Keywords
Transportation-Related	ride, driver, car, taxi, ojek, ojol, perjalanan, supir, transportasi, tarif, penumpang, trayek
Non-Transport Services	food, delivery, restaurant, order, eat, meal, drink, grocery, mart, service, package, send, courier, makanan, antar, pesanan, minuman, gofood, grabfood, belanja, paket, barang, kirim, kurir

### 4.2 Data preparation

We followed predetermined labeling guidelines to annotate subjectivity within our dataset. The labeling guidelines were created based on the definition of subjectivity and objectivity in [19]. Where objective texts present factual information and evidence. In contrast, subjective texts reflect personal opinions, emotions, and value judgments, frequently appearing in opinion articles and creative writing. For most reviews, which might often contain both objective and subjective elements in one review, we follow the previous study [20] to label them. If the primary intent of the sentence was to convey factual information, it was labelled as Objective; otherwise, it was labelled as Subjective. Based on those statements, we construct our labeling guideline tailored to the need for ride-hailing app reviews. The presence of opinions, emotions, or imprecise expressions characterized subjective reviews.

In contrast, objective reviews contained measurable and verifiable facts. Table 4 shows the labeling guideline, tailored explicitly for the Bahasa Indonesia reviews in the ride-hailing app field. This guideline plays a massive role in accurately categorizing subjective content in users’ reviews.

**Table 4:** Labeling guideline for subjectivity classification.

Category	Indicators
**Subjective**	- Opinions, emotions, and personal dissatisfaction. - Imprecise terms or generalizations (e.g., "mahal", "lama", "jauh" without numbers). - Use of vague frequency words (e.g., "sering", "suka", "kadang-kadang"). - Frustration or disappointment without evidence of system failure. - Personal perceptions rather than systemic issues. - Subjective comparisons without measurable data.
**Objective**	- Verifiable facts or specific events. - Reports of app malfunctions (e.g., crash, GPS error, payment issue). - Fare miscalculations or discrepancies between the driver and the app. - Driver misconduct with clear, verifiable context (e.g., overcharging, unfair cancellation, threatening behavior). - Quantifiable data tied to technical/system errors (e.g., "menunggu 1 jam karena aplikasi error"). - Systemic failures or observable technical problems.

This focus ensures that the subjectivity detection conducted using our dataset is more closely aligned with the linguistic and cultural context of Bahasa Indonesia, thereby improving the accuracy and relevance of the research findings within this specific domain. As our dataset exclusively consists of reviews written in Bahasa Indonesia, no additional language identification was necessary after filtering. To support the subjectivity detection task, we added a 'Label' column to indicate the classification of each review as either 'Subjective' or 'Objective.' Each review was manually annotated based on a predefined labeling guideline, emphasising the presence of opinions, emotions, or information. Reviews that could not be distinctly classified, due to containing a strong mixture of subjective and objective elements, were excluded to maintain a focus on clearly defined cases.

To ensure the reliability of manual annotation, a subset of 134 reviews (approximately 10 % of the dataset) was independently labelled by two annotators to measure inter-annotator agreement (IAA). Because only two annotators were involved, Cohen’s Kappa was chosen as the appropriate metric.

Key results include:

• Initial Cohen’s Kappa score: 0.283, reflecting a fair level of agreement, as interpreted from the scale proposed by Landis and Koch.
• Raw agreement rate: 74.62 %, with 34 instances of disagreement.
• Refinement of guidelines: Following discussions and refinements to the annotation guidelines, the agreement rate increased to 96.27 %, with five unresolved cases removed from the dataset.

After this calibration phase, the remaining 1213 reviews (90 % of the dataset) were annotated independently. With results showing:

• Cohen’s Kappa score: 0.694, indicating substantial agreement.
• Overall agreement rate: 88.62 %, with 11.38 % disagreement.
• Disagreements: Resolved through discussion, with only four unresolved cases removed.
• Final labeled dataset: 1338 reviews, with 378 reviews (28.25 %) classified as objective and 960 reviews (71.75 %) classified as subjective, now serving as the ground truth for further analysis.

The substantial improvement in inter-annotator agreement throughout the annotation process underscores the reliability and consistency of our manual labeling efforts. This approach ensures that the final labelled dataset, comprising 1338 reviews, serves as a robust ground truth for further analysis, enhancing the validity of the subsequent findings in subjectivity detection.

### 4.3 Exploratory analysis

**Fig. 4:**
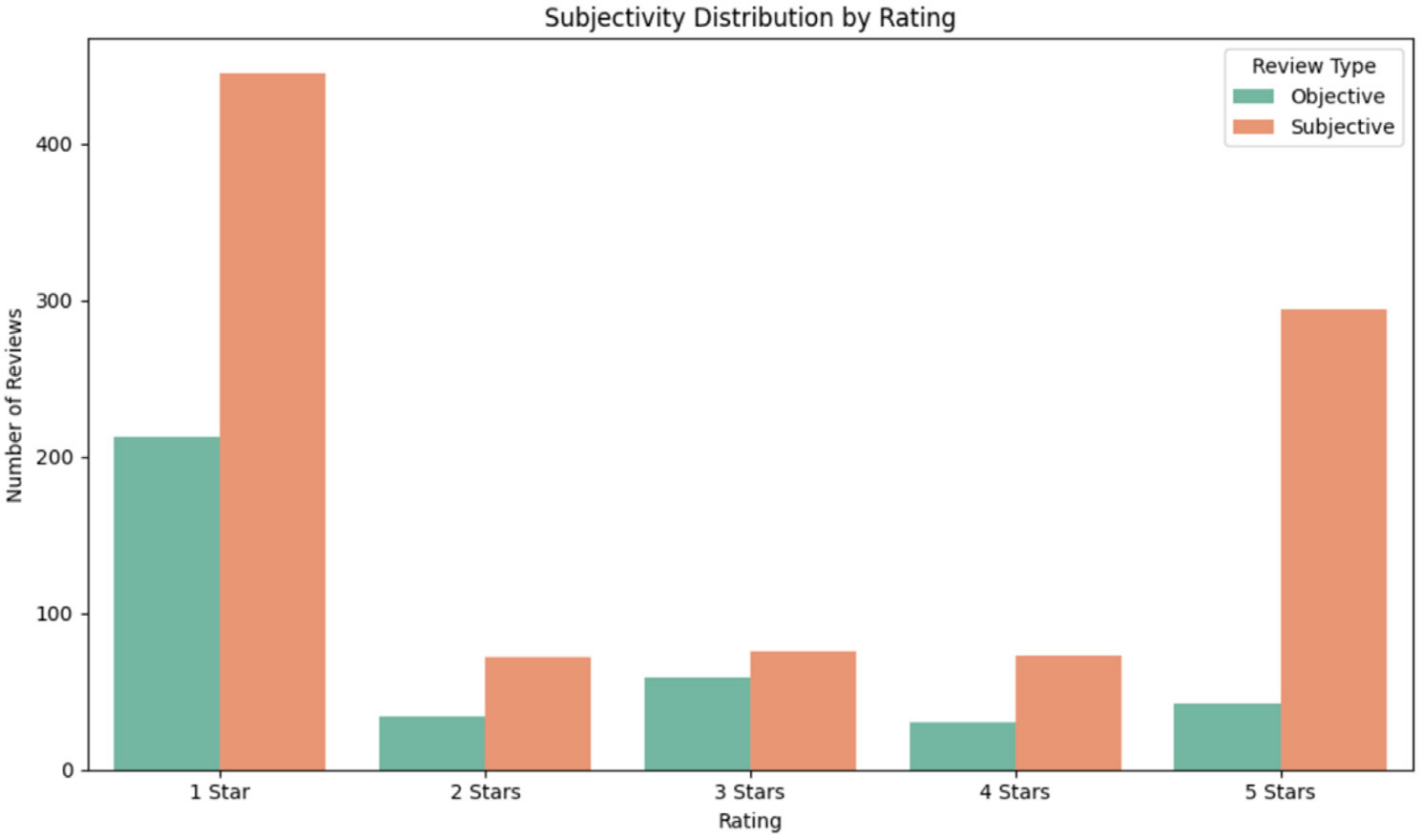
Rating distribution of the dataset, generated using Python and the Seaborn and Matplotlib libraries.

Recognising the potential influence of review ratings in understanding user expression, we visualised the distribution of review ratings across subjective and objective reviews. As shown in Fig. 4, subjective reviews were more frequent across all rating levels, with the highest counts observed at the 1-star rating. Objective reviews also showed their highest frequency at the 1-star rating, followed by moderate occurrences in other categories. This figure is included solely to demonstrate one possible analytical use of the dataset.

**Fig. 5:**
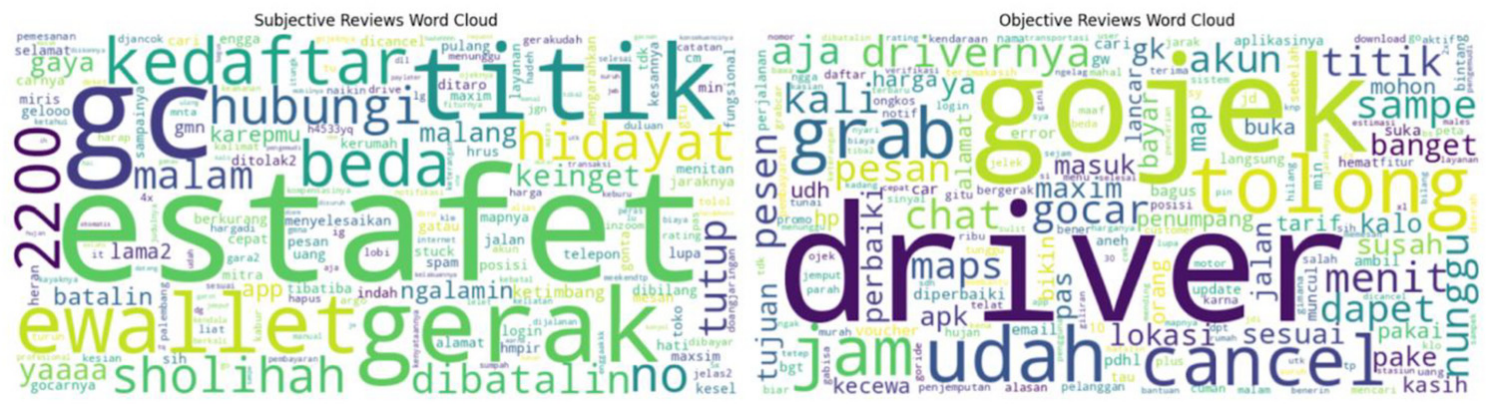
Word cloud created using the Python wordcloud library without stopword filtering.

We also generated a word cloud (Fig. 5) to illustrate commonly appearing words in subjective and objective reviews. The word cloud was created using the Python wordcloud library without stopword removal, allowing all terms to appear according to their frequency. Fig. 4, Fig. 5 are intended only as examples of how the dataset may be explored, rather than as analytical findings.

In subjective reviews, the most prominent words included "estafet", "gc", "titik", and "gerak", reflecting personal opinions and specific experiences. Words like "ewallet", "beda", and "malang" further emphasize emotional expressions or vague impressions often found in subjective feedback. For example, terms such as "no", "yaaaa", and "hidayat" are indicative of users sharing feelings or evaluations that go beyond straightforward descriptions, which aligns with the typical nature of subjective reviews.

On the other hand, objective reviews featured more functional or factual terms, such as "driver", "gojek", "cancel", and "jam". These terms point to concrete elements of the ride-hailing service, focusing on the mechanics of the service experience. While there is some overlap in service-related terms between both categories, the overall tone of objective reviews remains more descriptive and focused on factual aspects of the user experience. Words like "titik" (point), "pesan" (order), and "menit" (minutes) were commonly used to provide objective details about the service or the transaction. These examples show differences that may be explored by researchers using this dataset.

## Limitations

The dataset used in this study does not encompass all ride-hailing platforms or review sources. It is limited to publicly available user reviews from specific app stores, primarily focusing on popular services such as Gojek and Grab. As a result, the findings may not fully generalise to lesser-known or region-specific transportation apps. Additionally, while manual labeling was employed to ensure quality in subjectivity classification, the process is inherently subjective and may introduce labeling bias. Another limitation is the lack of demographic or contextual metadata, such as user location or trip type, which could further influence reviews' subjective or objective nature. Thus, although the dataset offers valuable insights into user expression patterns in ride-hailing reviews, it may not represent the full spectrum of user behaviour across different platforms and use cases.

## Ethics Statement

The authors collected all data used in this study by scraping publicly available ride-hailing app reviews. The dataset was anonymised to protect users' privacy, and sensitive information such as user ID was redacted to ensure confidentiality. The research complies with privacy standards and data protection regulations. No human subjects or animals were involved in this study, and the data were solely used for academic purposes, not for profit. The reviews used in this study are limited to public information available on Google Play stores and do not include data from private or social media platforms. The authors confirm adherence to the ethical standards of relevant data use and research guidelines, ensuring responsible and transparent data handling.

- Copyright: Our project is committed to respecting copyright and intellectual property rights. We adhere to the legal and ethical standards for using content from the Google Play Store or applications. We only collect publicly available data and do not engage in activities that infringe on copyright laws. Any data we collect is used for research and analysis purposes only.
- Privacy: We prioritize user privacy and adhere to all relevant privacy laws and guidelines. When collecting data from the Google Play Store or applications, we ensure that no personally identifiable information is gathered. We focus solely on aggregating publicly available information while respecting user privacy and consent. Our data collection methods are in compliance with the applicable privacy policies and regulations.
- Web Scraping Policies: We are aware of the importance of respecting web scraping policies. For platforms like Google Play Store, we follow any specific scraping policies and guidelines they have in place. We use standard web scraping techniques and ratelimit our requests to minimize any impact on the platform. Our goal is to collect data responsibly, ensuring the integrity of the Google Play Store and applications.
- Terms of Service: We are committed to adhering to the Terms of Service (ToS) of the websites and platforms we interact with. This includes the Google Play Store and any applications we may access. We review and monitor the ToS regularly to ensure that our data collection practices remain compliant. If there are any specific scraping policies outlined in the ToS, we follow them closely.

## CRediT Author Statement

**Violeta Arifin**: Conceptualization, Methodology, Data curation, Software, Visualization, Writing - original draft, Writing - review & editing; **Yuriashi Adelia Putri**: Data collection, Formal analysis, Data curation, Software, Visualization, Writing - original draft, Writing - review & editing; **Richard Wiputra**: Supervision, Software, Writing – review and editing

## Data Availability

Mendeley DataSubRide: Subjectivity Detection in Ride-Hailing App Reviews (Original data).
